# Risk Factors and Prevention Strategies of Nosocomial Infection in Geriatric Patients

**DOI:** 10.1155/2019/6417959

**Published:** 2019-02-25

**Authors:** Yajie Li, Liqun Ren, Jihong Zou

**Affiliations:** Geriatrics Department, Zhongda Hospital Southeast University, No. 87, Dingjiaqiao, Nanjing, China

## Abstract

**Introduction:**

To investigate the risk factors of nosocomial infections (NIs) in geriatric department and the effectiveness of the proposed prevention strategy.

**Methodology:**

We studied 3370 cases of elderly patients who were hospitalized more than 48 hours from January 2015 to December 2017 in the Geriatrics Department of Zhongda Hospital, Southeast University. In order to reduce the infection rate, nutritional risk screening (NRS 2002) was used to evaluate the nutritional status of the patients; enteral nutritional suspension (TPF-FOS) was provided to the patients who were assessed to be necessary.

**Results:**

Before prevention strategy was taken, the nosocomial infection rate was 3.3% (80 among 2413 patients) in our department. The most frequent NIs were pneumonia (60 cases) followed by urinary tract infection (30 cases). It is worth noting that the elderly patients are often associated with multiple infections: in our study, 15 patients have pneumonia and UTI at the same time. After prevention strategy was taken, the nosocomial infection rate reduced to 1.15% (11 among 957 patients) in our department.

**Conclusions:**

NIs are common in elderly patients. The improvement of the nutritional status of patients is effective in reducing the risks of NIs.

## 1. Introduction

As the largest developing country in the world, China is now facing a serious population aging problem: by 2020, the elderly population is expected to reach 248 million and the aging level to 17.17%, of which 30.67 million will be aged 80 and above [1]. Therefore, it is urgent to improve the evaluation and treatment for elderly patients. However, elderly people are susceptible to infections due to hypoimmunity [2], which is caused by malnutrition, comorbidity, decreased antibody production, delays in their cellular and humoral immune responses, etc. [3]. Especially when elderly patients are hospitalized after 48 hours and exposed to invasive procedures in an environment abundant with virulent and antibiotic-resistant pathogens, the risk of nosocomial infections (NIs) is even higher [4, 5].

Despite the fact that the risk for elderly patients to get NIs is obviously higher, the risk factors in geriatric patients are still not well known [6, 7]. The aim of this study is to determine the risk factors leading to NI development and discuss the effectiveness of nutrition status improvement in prevention of NIs for elderly patients.

## 2. Methodology

### 2.1. Study Design

A retrospective analysis was conducted. The distribution of the patient's infection and the pathogenic microorganisms of the infections identified as NIs in the elderly patients are carefully recorded. The age of the patient, sex, length of hospital stay, whether the patient was in bed for a long time, whether the patient combined with chronic diseases, hypoproteinaemia, dysphagia, invasive procedure, etc., were analyzed in detail.

Malnutrition was considered to be one of the main causes of NIs. On admission, based on NRS 2002, the patients were scored according to weight, albumin level, and disease status. If the score is <3, no intervention will be taken. If the score is ≥3, daily diet will be supplied according to the required daily calorie calculation formula (energy = 25 kCal/kg·d); also, enteral nutrition suspension (TPF-FOS) [8] is provided to increase energy. Comparative analysis was done between before and after the prevention strategy was taken.

### 2.2. Clinical Data

Zhongda Hospital, Southeast University, has a database which includes all the medical reports and a real-time hospital infection monitoring system. The data were collected between January 2015 to December 2017 in the Geriatrics Department based on the database and the system.

Before the NI prevention strategy was taken, 2413 hospitalized patients (2015.1–2017.5) including 1294 males and 1119 females were investigated; the patients' age ranged from 65 to 101 years, and the mean age was 82 ± 7.83 years. After taking prevention strategy to improve the nutritional status of the patients, 957 hospitalized patients (2017.6–2017.12) including 506 males and 451 females were investigated; the patients's age ranged from 65 to 103 years, and the mean age was 84 ± 9.26 years.

### 2.3. Diagnosis Criteria

The diagnosis criteria for nosocomial infection is determined in accordance with the criteria for diagnosis of nosocomial infection (trial) promulgated by the Ministry of Health of the People's Republic of China in November 2001 [9].

Nutritional risk screening tool: Nutritional Risk Screening 2002 (NRS 2002) [10], which was recommended by the European Society for Clinical Nutrition and Metabolism (ESPEN), was used to evaluate the nutritional status of the elderly patients.

### 2.4. Statistical Analysis

The Statistical Package for the Social Sciences version10.0 (SPSS) was used for statistical evaluation: *T*-test, chi-square tests for univariate, and logistic regression analysis for multivariate were calculated for descriptive statistics. A *p* value < 0.05 was considered statistically significant.

## 3. Results

In this study, 3370 patients (age larger than 65 years old) were hospitalized more than 48 hours in the Geriatric Department.

Before nutritional risk screening was taken, NIs were detected in 80 patients among 2413 patients; the infection rate was 3.3% (80 in 2413 patients). The most frequent distribution is pneumonia (60 cases of infection in 80 patients), followed by urinary tract infection (30 cases of infection). Among them, 15 patients have pneumonia and urinary tract infection at the same time. The details of NIs' distribution according to systems are listed in Table 1.

As shown in Table 2, all 80 patients were sent for examination of pathogens, and 58 strains were detected. Among them, 8 strains were Gram-positive bacteria, dominant with *Staphylococcus*; 36 strains were Gram-negative bacteria dominant with *Pseudomonas aeruginosa*, *E. coli*, and *Proteus mirabilis*; 14 strains were fungi dominant with *Candida albicans* and *Candida glabrata*.

Comparison from clinical features between the two samples (sample_1 are 2413 patients who were not given prevention, while sample_2 are 957 patients who were scored and provided with enteral nutritional suspension) are listed in Tables 3 and 4. No statistical significance can be found. After providing enteral nutritional suspension (TPF-FOS) to the patients according to the screening results, the infection rate for sample_2 was reduced to 1.15% (11 in 957 patients). Statistical significance is observed in Table 5, and the 95% CI is 0.180∼0.640.

Risk factors of NIs such as long-term bed (>30 days of hospitalization), combined with chronic diseases, existence of invasive procedures, malnutrition (score ≥ 3), dysphagia, and so on were taken into consideration. The univariate analysis results showed that long-term bed, malnutrition, invasive procedures, dysphagia, and length of stay are all possible factors in developing NIs (see Table 6).

Multivariate analysis of the risk factors is shown in Table 7. The probability of NIs based on long-term bed, nutrition status, dysphagia, invasive procedure, and length of stay is less than 0.01. Therefore, they can be used to predict NIs in our department.

## 4. Discussion

In this study, before infection prevention strategy was taken, the incidence of NIs is 3.3%, and the hospitalized duration in our Geriatric Department for per patient is 11 days. The incidence rate is similar to [11] (2.49 per 1000 patient days). The mortality rate is 5% (4 patients died among 80 nosocomial infected patients), while during the same period, for elderly patients without NIs, the mortality rate is 2.27% (53 patients died among 2333 patients). The high risk of NIs for elderly patients indicates that the infection control measures are necessary.

The distribution of NIs in geriatric patients was examined, and the most common infection in our hospital is pneumonia, which is the same as [6, 12, 13]. However, compared to the former research studies, the frequency of our study (63.15%) is a little too high compared to other developing or developed countries (43% in [2] and 45% in [4]); this may have some relation with the nowadays bad air condition in China. Since the mortality of nosocomial pneumonia in Asian countries is very high [14], lots of attention should be paid.

Urinary tract infection is one of the most common nosocomial infections around the world [2, 15–17], and UTI is the secondary common infection in our study. Since UTI is usually the result of placing and removing indwelling urinary catheters; more attention should be paid continuously.

Totally, 58 strains of pathogens were isolated, of which 36 (62.07%) were Gram-negative bacteria, 8 (13.79%) were Gram-positive bacteria, and 14 (24.14%) were fungi; *Pseudomonas aeruginosa*, *E. coli*, and *Proteus mirabilis* were dominant among the Gram-negative bacteria; *Staphylococcus* was the predominant species of the Gram-positive bacteria; *Candida albicans* and *Candida glabrata* were dominant among the fungi. The distribution of the microorganisms is similar to the research in our country [18]. Since in geriatric department, common antibiotic treatment and routine treatment are difficult to get rid of the pathogen completely. Newly developed disinfecting techniques [19, 20], like UVC LEDs can be used together to reduce bacterial contamination and against common microorganisms.

Geriatric Department in our hospital has a higher NI rate than other units. This is related with the older age and relative longer duration of hospitalization of our patients. Our research also revealed that invasive procedures, malnutrition, long-term bed, dysphagia, and length of stay are all statistically significant in causing NIs. If the elderly patients stay in the hospital for more than 30 days, the risk of infection will increase greatly; this is because patients with long hospitalization are prone to cross infection [21]. Dysphagia is another significant influence factor for NIs [22], since patients with dysphagia are prone to eating cough and gastrointestinal dysfunction, which will lead to food reflux in the stomach and lung infection. Patients with dysphagia should be given a gastric tube, and the head of the bed needs to be raised while patients are sleeping. Long-term bed is also a risk factor, for bedridden patients; turning over frequently or real-time monitoring system maybe necessary [23]. These specific factors should draw enough attention in the development of infection control measures.

Elderly patients are at high risk of infection, and malnutrition maybe one factor with particularly importance. The result of our research is similar to the cases in the USA [2] and our country [21, 22]. Also in [2], it is uncertain whether a specially designed program for preventing infections in elderly patients is required. But through our research, after studying two similar samples, using already existing nutrition risk screening tools such as NRS 2002 to score the patients, and improving the nutrition status of the elderly patients with enteral nutritional suspension (TPF-FOS), the NIs in elderly patients can be effectively prevented. So in order to reduce the infection rate, poor nutrition of elderly patients should be controlled; procedures such as assessing the nutritional status of the patient and strengthening gastrointestinal nutrition, etc., are perhaps the most effective methods at present.

## 5. Conclusion

In conclusion, NIs are common in geriatric department, and the most common infections are pneumonia and urinary tract infection. Considering the high risks, infection control efforts for pneumonia and UTI should continue to be an important program priority. For the infected patients, pathogens include Gram-positive bacteria, Gram-negative bacteria, and fungi. The empirical treatment decisions in geriatric department can be based on those pathogens. Through our research, long-term bed, malnutrition, invasive procedures, dysphagia, and length of stay are all independent risk factors for NIs in elderly patients. The improvement of nutrition status is important and perhaps one of the few feasible strategies that can be taken in infection control and has been proved to be effective in this paper.

Because this paper is a retrospective analysis, the evaluation of nutritional status before and after nutrition intervention is not comprehensive enough, which will be improved in the future research.

## Figures and Tables

**Table 1:** Distribution of NIs.

Nosocomial infections	Infected cases (*N* = 95)
Pneumonia	60 (63.15%)
Urinary tract infection	30 (31.58%)
Skin and soft tissue infection	2 (2.12%)
Gastrointestinal system	1 (1.05%)
Oral infection	1 (1.05%)
Intraperitoneal infection	1 (1.05%)

**Table 2:** NIs developed by microorganism.

Microorganisms	Bacterial species (*N*/*N* total, %)
Gram-positive bacteria	*Staphylococcus* (5/8, 62.5%)
	Excrement enterococcus (3/8, 37.5%)

Gram-negative bacteria	*Pseudomonas aeruginosa* (7/36, 19.4%)
	*E. coli* (4/36, 11.1%)
	*Proteus mirabilis* (4/36, 11.1%)
	*Acinetobacter baumannii* (3/36, 8.3%)
	*Klebsiella pneumoniae* (3/36, 8.3%)
	*Stenotrophomonas maltophilia* (3/36, 8.3%)
	Others (12/36, 33.3%)

Fungi	*Candida albicans* (5/14, 35.7%)
	*Candida glabrata* (6/14, 42.9%)
	*Candida tropicalis* (2/14, 14.3%)
	*Candida krusei* (1/14, 7.1%)

**Table 3:** Analysis of two samples before and after improving of nutrition status.

	Age	*p* value (*p* < 0.05)
Sample_1	82 ± 7.83	0.716
Sample_2	84 ± 9.26	

**Table 4:** Analysis of two samples before and after improving of nutrition status.

Variate	Two samples	Number/total number	*χ* ^2^	*p* value (*p* < 0.05)
Sex (male)	Sample_1	1294/2413	0.156	0.693
	Sample_2	506/957		

Long-term bed	Sample_1	212/2413	0.572	0.450
	Sample_2	92/957		

Nutrition scores (≥3)	Sample_1	132/2413	1.037	0.309
	Sample_2	61/957		

Dysphagia	Sample_1	174/2413	0.044	0.834
	Sample_2	71/957		

Invasive procedure	Sample_1	672/2413	2.872	0.090
	Sample_2	239/957		

Length of stay (≥30 day)	Sample_1	77/2413	3.599	0.058
	Sample_2	19/957		

Combined with chronic disease	Sample_1	1259/2413	2.646	0.104
	Sample_2	529/957		
	Sample_2	11/957		

**Table 5:** Analysis of two samples before and after improving of nutrition status.

	With NIs (number/total number)	*χ* ^2^	*p* value (*p* < 0.05)
Sample_1	80/2413	12.235	<0.05
Sample_2	11/957		

**Table 6:** Analysis of risk factors of nosocomial infection in geriatric patients.

Personal characteristics		Number	Infected patients	*χ* ^2^	*p* value (*p* < 0.05)
Sex	Male	1800	48	0.017	0.897
	Female	1570	43		

Long-term bed	Yes	304	52	263.896	<0.01
	No	3066	39		

Combined with chronic disease	Yes	1788	49	0.023	0.878
	No	1582	42		

Nutrition scores	<3	3177	69	58.959	<0.01
	≥3	193	22		

Dysphagia	Yes	245	15	11.777	<0.01
	No	3125	76		

Invasive procedure	Yes	911	37	8.804	<0.01
	No	2459	54		

Length of stay (day)	≥30	96	19	109.863	<0.01
	<30	3274	72		

**Table 7:** Multivariate analysis of risk factors of nosocomial infection in geriatric patients.

Variate	Wald	*p* value	OR	95% CI
Long-term bed	5.181	0.023	2.484	1.135∼5.473
Nutrition scores (≥3)	8.844	<0.01	3.463	1.527∼7.852
Dysphagia	16.898	<0.01	7.909	2.951∼21.202
Invasive procedure	10.567	<0.01	1.181	1.064∼1.507
Length of stay (day)	117.953	<0.01	94.565	41.608∼214.921

## Data Availability

The data used to support the findings of this study are available from the corresponding author upon request.
